# TCR signaling and cellular metabolism regulate the capacity of murine epidermal γδ T cells to rapidly produce IL-13 but not IFN-γ

**DOI:** 10.3389/fimmu.2024.1361139

**Published:** 2024-02-28

**Authors:** Atsuko Ibusuki, Kazuhiro Kawai, Ayano Nitahara-Takeuchi, Rafael J. Argüello, Takuro Kanekura

**Affiliations:** ^1^ Department of Dermatology, Kagoshima University Graduate School of Medical and Dental Sciences, Kagoshima, Japan; ^2^ Department of Dermatology, Kido Hospital, Niigata, Japan; ^3^ Division of Dermatology, Niigata University Graduate School of Medical and Dental Sciences, Niigata, Japan; ^4^ Aix Marseille Université, CNRS, INSERM, CIML, Centre d’Immunologie de Marseille-Luminy, Marseille, France

**Keywords:** mouse, intraepithelial lymphocytes, cytokine, T-cell receptor, mTORC1

## Abstract

Resident epidermal T cells of murine skin, called dendritic epidermal T cells (DETCs), express an invariant γδ TCR that recognizes an unidentified self-ligand expressed on epidermal keratinocytes. Although their fetal thymic precursors are preprogrammed to produce IFN-γ, DETCs in the adult epidermis rapidly produce IL-13 but not IFN-γ early after activation. Here, we show that preprogrammed IFN-γ-producing DETC precursors differentiate into rapid IL-13 producers in the perinatal epidermis. The addition of various inhibitors of signaling pathways downstream of TCR to the *in vitro* differentiation model of neonatal DETCs revealed that TCR signaling through the p38 MAPK pathway is essential for the functional differentiation of neonatal DETCs. Constitutive TCR signaling at steady state was also shown to be needed for the maintenance of the rapid IL-13-producing capacity of adult DETCs because *in vivo* treatment with the p38 MAPK inhibitor decreased adult DETCs with the rapid IL-13-producing capacity. Adult DETCs under steady-state conditions had lower glycolytic capacity than proliferating neonatal DETCs. TCR stimulation of adult DETCs induced high glycolytic capacity and IFN-γ production during the late phase of activation. Inhibition of glycolysis decreased IFN-γ but not IL-13 production by adult DETCs during the late phase of activation. These results demonstrate that TCR signaling promotes the differentiation of IL-13-producing DETCs in the perinatal epidermis and is needed for maintaining the rapid IL-13-producing capacity of adult DETCs. The low glycolytic capacity of adult DETCs at steady state also regulates the rapid IL-13 response and delayed IFN-γ production after activation.

## Introduction

γδ T cells represent a minor population of T cells in adult blood and peripheral lymphoid organs but are enriched in epithelial tissues (1, 2). In mice, different epithelial tissues are populated by distinct γδ T-cell subsets defined by the usage of specific TCR Vγ regions (3). These epithelial γδ T-cell subsets develop in waves based on ordered *Vγ* gene rearrangement in the fetal and neonatal thymus and are home to specific tissues. Epithelial γδ T cells mediate stress surveillance and exert a distinct set of effector functions in a given tissue (4–8).

Unlike conventional αβ T cells, which differentiate into effector subsets during activation in peripheral lymphoid organs (9), most epithelial γδ T cells are preprogrammed during thymic development to either IFN-γ- or IL-17A-producing effector subsets that exhibit rapid, innate-like responses in the periphery (10–12). Recent studies have revealed the role of TCR signaling in the differentiation of effector subsets during thymic development. Ligand-induced strong TCR signaling is needed for the differentiation of IFN-γ-producing γδ T cells, whereas weaker TCR signaling supports the differentiation of IL-17A-producing cells (13–18). IFN-γ-producing γδ T-cell development also requires CD27 signaling, and mature IFN-γ-producing γδ T cells usually express CD27, while IL-17A-producing γδ T cells lack CD27 expression (14).

Resident epidermal T cells of murine skin, called dendritic epidermal T cells (DETCs), are prototypic epithelial γδ T cells that express an invariant Vγ3Vδ1 TCR (Garman nomenclature) (19). DETCs contribute to epidermal homeostasis, wound healing, IgE production, and tumor surveillance (7, 20–25). Although the ligand of the Vγ3Vδ1 TCR has not been identified, it is expressed on fetal thymic epithelial cells and stressed or transformed epidermal keratinocytes (26–29). Accumulating evidence suggests that low levels of the TCR ligand are constitutively expressed on keratinocytes at steady state (20, 30). DETC precursors develop as the first T cells in the fetal thymus. Thymic maturation of DETC precursors requires ligand-dependent TCR signaling and the thymic stromal determinants Skint1 and Skint2 (15, 31, 32). TCR signaling in DETC precursors in the fetal thymus promotes the upregulation of skin homing receptors needed for thymic export and skin homing (33–35), and DETC precursors migrate to the skin before birth. After seeding the epidermis in low numbers, DETCs massively proliferate until 2 to 6 weeks after birth, depending on the mouse strain (36, 37), and their numbers become stable by 8 weeks (27).

Ligand-selected mature Vγ3^+^ fetal thymocytes that have received strong TCR signaling express CD27 and *Tbx21*, which encodes T-bet, the master transcription factor of IFN-γ-producing cells, and rapidly produce IFN-γ following phorbol 12-myristate 13-acetate (PMA) and ionomycin stimulation (15). In contrast, DETCs in the adult epidermis do not express CD27 (38, 39) or produce IFN-γ within 4 hours after stimulation with PMA/ionomycin (22, 40) but do begin to produce IFN-γ 12 to 24 hours after stimulation *in vitro* (39, 41). This could be due to the hyporesponsive TCR signaling that occurs during DETC development (42). However, adult DETCs produce IL-13 upon short-term PMA/ionomycin stimulation (22). Therefore, Vγ3^+^ T cells appear to differentiate from preprogrammed IFN-γ producers into rapid IL-13 producers after thymic egress. This functional switch in DETCs sets them apart from other tissue-resident γδ T cells preprogrammed to produce IFN-γ, and the distinct kinetics of IL-13 and IFN-γ production by DETCs is important for DETC-mediated stress surveillance in the epidermis (22, 24). However, it remains unknown when, where, and how the functional switch of Vγ3^+^ T cells occurs.

We aimed to clarify the timing, location, and underlying mechanisms of the functional switch of Vγ3^+^ T cells. Here, we show that Vγ3^+^ T cells differentiate from preprogrammed IFN-γ producers into rapid IL-13 producers in the perinatal epidermis and that this differentiation is dependent on TCR signaling through the p38 mitogen-activated protein kinase (MAPK) pathway. We also show that the rapid IL-13-producing capacity of adult DETCs under steady-state conditions is maintained by continuous TCR signaling and cellular metabolism.

## Results

### Adult DETCs predominantly produce IL-13 during the early phase of activation

**Figure 1:**
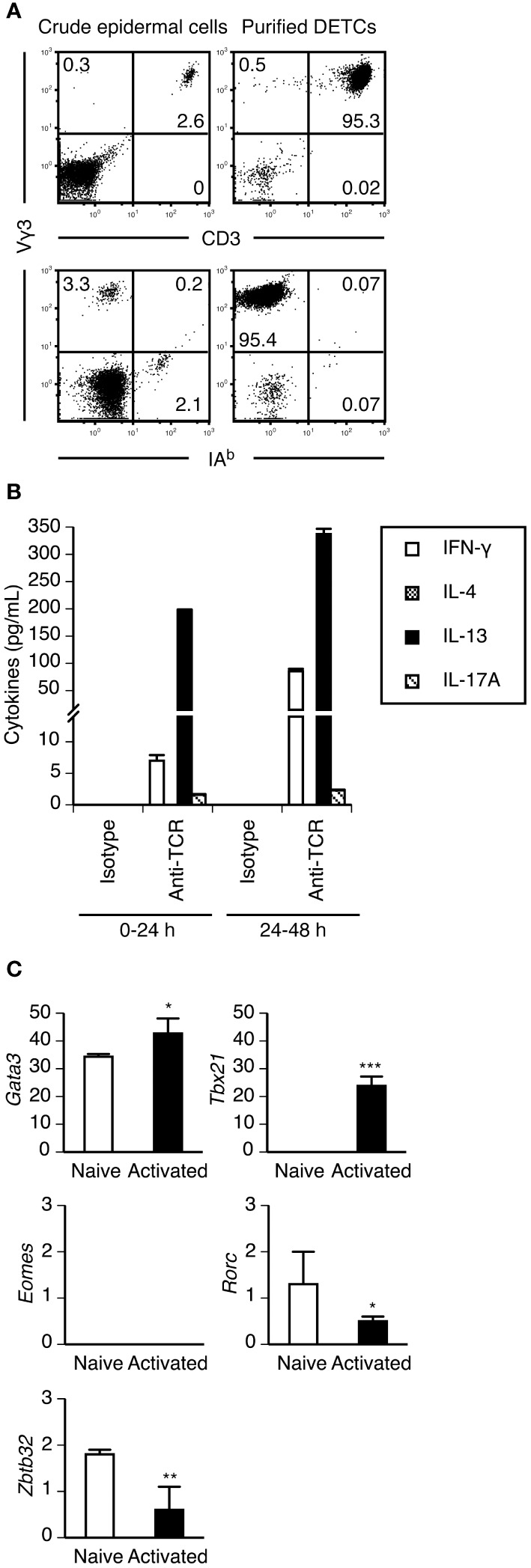
Purified adult DETCs predominantly produce IL-13 during the early phase of activation. **(A)** Crude epidermal cells isolated from adult ear skin and DETCs purified from epidermal cells by positive magnetic selection using anti-integrin β7 mAb were stained with the indicated mAbs. Quadrant settings were determined by staining with isotype control mAbs. The numbers denote the percentages of cells in the respective quadrants. Representative profiles from two independent experiments are shown. In each experiment, cells pooled from 3-4 mice were analyzed. **(B)** Purified DETCs were stimulated with immobilized isotype control or anti-TCR mAb. Culture supernatants were harvested and replaced with fresh culture medium 24 hours after the start of stimulation, and DETCs were stimulated for an additional 24 hours. Cytokine levels in the supernatants were determined by ELISA. Representative data from two independent experiments are shown as the mean and SD of triplicate cultures. **(C)** mRNA expression of the indicated transcription factors in purified DETCs without stimulation (naive) and purified DETCs stimulated with immobilized anti-TCR mAb for 24 hours (activated) was analyzed in triplicate by real-time RT–PCR. Representative data from two independent experiments are shown as the mean and SD. Significant differences compared with naive DETCs are denoted with asterisks (*P < 0.05, **P < 0.01, ***P < 0.001).

Adult DETCs produce IL-13 but not IFN-γ upon short-term PMA/ionomycin stimulation (22). To quantify cytokine levels secreted by adult DETCs upon TCR stimulation, we purified DETCs from adult ear epidermal cells without TCR ligation by positive magnetic selection using an anti-integrin β7 mAb. The purified DETCs were >95% pure Vγ3^+^ T cells and contained <1% non-Vγ3^+^ T cells and <1% IA^+^ Langerhans cells (**Figure 1A**). Although DETCs were reported to constitutively produce IL-13 at steady state (22), purified DETCs cultured without TCR stimulation did not secrete IL-13 or other cytokines (**Figure 1B**). The purified DETCs predominantly secreted IL-13 during the first 24 hours of TCR stimulation, and IFN-γ secretion increased after 24 hours. Consistent with a previous study (39), DETCs secreted a small amount of IL-17A upon TCR stimulation, but IL-4 secretion was not detected (**Figure 1B**).

RT–PCR analysis revealed that purified naive DETCs constitutively expressed *Gata3*, the master transcription factor of type 2 cytokine-producing cells, but *Tbx21* was expressed only after activation (**Figure 1C**). *Eomes*, which encodes eomesodermin that also regulates IFN-γ production in type 1 cytokine-producing T cells, was not expressed in DETCs even after activation (**Figure 1C**). Consistent with the IL-17A-producing capacity of a subpopulation of DETCs, the expression of *Rorc*, which encodes RORγt, the master transcription factor of IL-17A-producing cells, was detected in naive DETCs (**Figure 1C**). The lack of IL-4 production by DETCs might be explained by the constitutive expression of *Zbtb32* (**Figure 1C**), which encodes ZBTB32 (Repressor of GATA, ROG) that represses *Il4* but not *Il13* gene activation in type 2 CD8^+^ cytotoxic T lymphocytes (43).

These results confirmed that under steady-state conditions, DETCs in the adult epidermis predominantly produce IL-13 during the early phase of activation. However, DETCs can produce IFN-γ during the later phase of activation. Therefore, unlike type 2 CD4^+^ helper T cells (44), the IFN-γ-producing capacity of DETCs would not be repressed by stable epigenetic modifications, as is the case for IL-17A-producing CD27^-^ γδ T cells, which can produce IFN-γ in a certain inflammatory microenvironment (45).

### Vγ3^+^ T cells lose CD27 expression immediately after migration to the dermis

**Figure 2:**
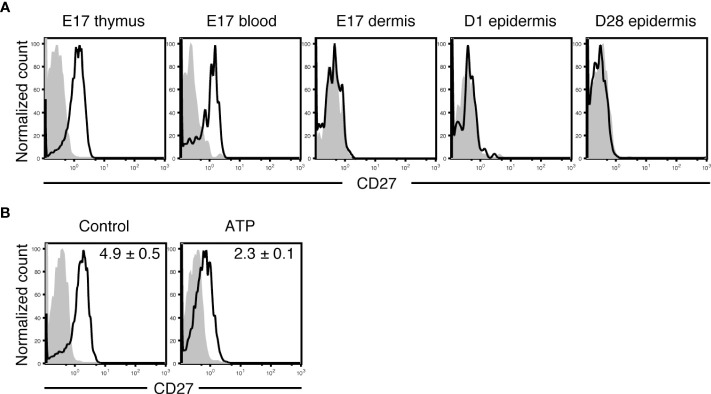
Vγ3^+^ T cells lose CD27 expression immediately after migration to the dermis. **(A)** Cells isolated from the indicated tissues at the indicated embryonic (E) or postnatal day (D) were stained with anti-Vγ3 mAb and anti-CD27 or isotype control mAb. Vγ3^+^ T cells were gated, and CD27 expression is shown as open histograms. Shaded histograms indicate cells stained with isotype control mAb. Each profile is representative of three independent experiments. In each experiment, cells pooled from 3-8 fetuses and 1-2 postnatal mice were analyzed. **(B)** E17 fetal thymocytes were treated with PBS (control) or ATP for 30 minutes and stained with anti-Vγ3 mAb and anti-CD27 or isotype control mAb. Vγ3^+^ T cells were gated, and CD27 expression is shown as open histograms. Shaded histograms indicate cells stained with isotype control mAb. Representative profiles from three independent experiments are shown. In each experiment, cells pooled from 9-10 fetuses were analyzed. Relative mean fluorescence intensity (MFI) was determined as (geometric MFI of CD27)/(geometric MFI of isotype control) and is shown in each panel as the mean and SEM (n = 3). Compared with the control, ATP treatment significantly diminished CD27 expression (*P* < 0.01).

**Figure 3:**
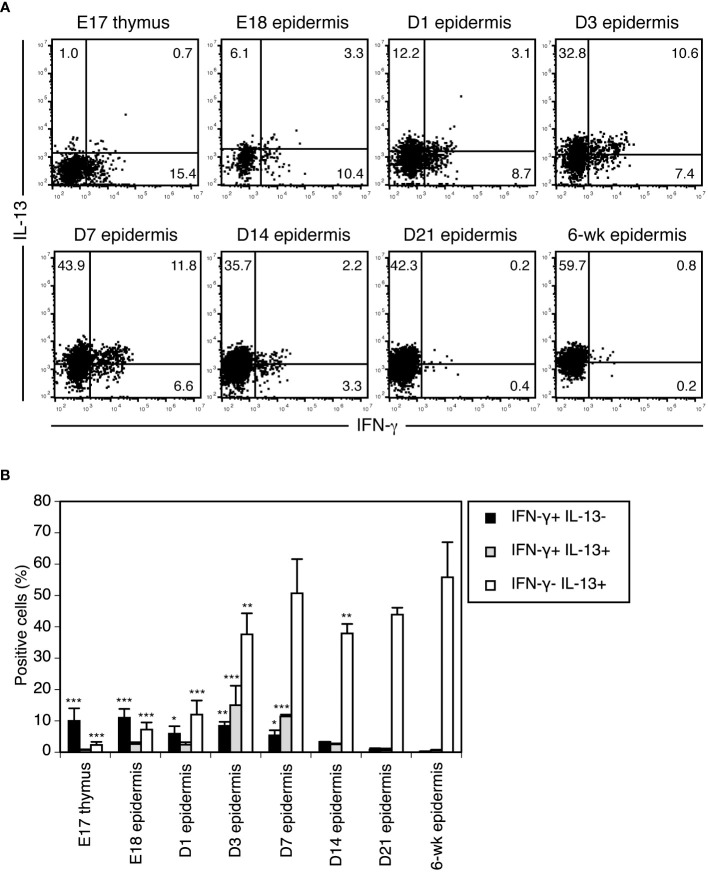
Vγ3^+^ T cells differentiate from preprogrammed IFN-γ producers into rapid IL-13 producers in the perinatal epidermis. **(A)** Cells isolated from the thymus or epidermis at the indicated time points were stimulated with PMA/ionomycin for 4 hours. Vγ3^+^ T cells were gated, and intracellular IFN-γ and IL-13 production was analyzed by flow cytometry. Quadrant settings were determined by staining with isotype control mAbs. The numbers denote the percentages of cells in the respective quadrants. Each profile is representative of three independent experiments. In each experiment, cells pooled from 5-8 fetuses and 1-3 postnatal mice were analyzed. **(B)** Quantification of **(A)**. Data are expressed as the mean and SEM (n = 3). Significant differences compared with the epidermis at 6 weeks are denoted with asterisks (*P < 0.05, **P < 0.01, ***P < 0.001).

Similar to embryonic day 17 (E17) mature Vγ3^+^ fetal thymocytes, circulating Vγ3^+^ T cells in the blood at E17 expressed CD27 (**Figure 2A**). As E17 fetal dermal Vγ3^+^ T cells did not express CD27 (**Figure 2A**), Vγ3^+^ T cells lost CD27 expression immediately after entering the dermis. A candidate that induces CD27 downregulation on Vγ3^+^ T cells is extracellular ATP, which would be abundant in the perinatal skin, because CD27 on T cells is rapidly shed and downregulated upon treatment with ATP (46). ATP treatment of E17 Vγ3^+^ fetal thymocytes resulted in rapid downregulation of CD27 (**Figure 2B**). As Vγ3^+^ T cells in day 1 (D1) neonatal epidermis that lacked CD27 expression (**Figure 2A**) could produce IFN-γ (**Figure 3**), CD27 expression and IFN-γ-producing capacity were not perfectly correlated in Vγ3^+^ T cells.

### Vγ3^+^ T cells differentiate from preprogrammed IFN-γ producers into rapid IL-13 producers in the perinatal epidermis

To clarify when and where Vγ3^+^ T cells functionally switch from preprogrammed IFN-γ producers to rapid IL-13 producers, we analyzed cytokines produced by Vγ3^+^ T cells upon short-term stimulation during ontogeny. Vγ3^+^ T cells in the E18 fetal epidermis still predominantly produced IFN-γ when stimulated with PMA/ionomycin for 4 hours (**Figure 3**). While IFN-γ-producing cells gradually decreased after birth, IL-13-producing cells gradually increased (**Figure 3**). As cells producing both IFN-γ and IL-13 transiently appeared in the neonatal epidermis (**Figure 3**), Vγ3^+^ T cells differentiated from preprogrammed IFN-γ producers into rapid IL-13 producers via intermediate IFN-γ/IL-13 producers during this period in the epidermis.

### Epidermal T cells expressing TCRs that recognize the self-ligand on epidermal keratinocytes predominantly produce IL-13

Thymic maturation of Vγ3^+^ T cells to IFN-γ-producing cells requires TCR signaling. As the proliferation of Vγ3^+^ T cells in the perinatal epidermis also relies on TCR signaling (47–49), we hypothesized that TCR signaling also induces the IL-13-producing capacity of Vγ3^+^ T cells in the perinatal epidermis.

To determine the role of TCR signaling in the induction of the IL-13-producing capacity of epidermal T cells, we analyzed cytokines produced by resident epidermal T cells of adult TCR δ-chain-deficient *Tcrd* ^-/-^ mice and TCR Vδ1-chain-deficient *Tcrd-V1* ^-/-^ mice. In *Tcrd* ^-/-^ mice lacking all γδ T cells, the epidermal niches of DETCs are replaced by αβ T cells, but the αβ TCRs expressed on these epidermal T cells cannot recognize the self-ligand on epidermal keratinocytes (27, 30). In contrast, epidermal T cells of *Tcrd-V1* ^-/-^ mice express diverse γδ TCRs that can recognize the self-ligand on epidermal keratinocytes (50).

**Figure 4:**
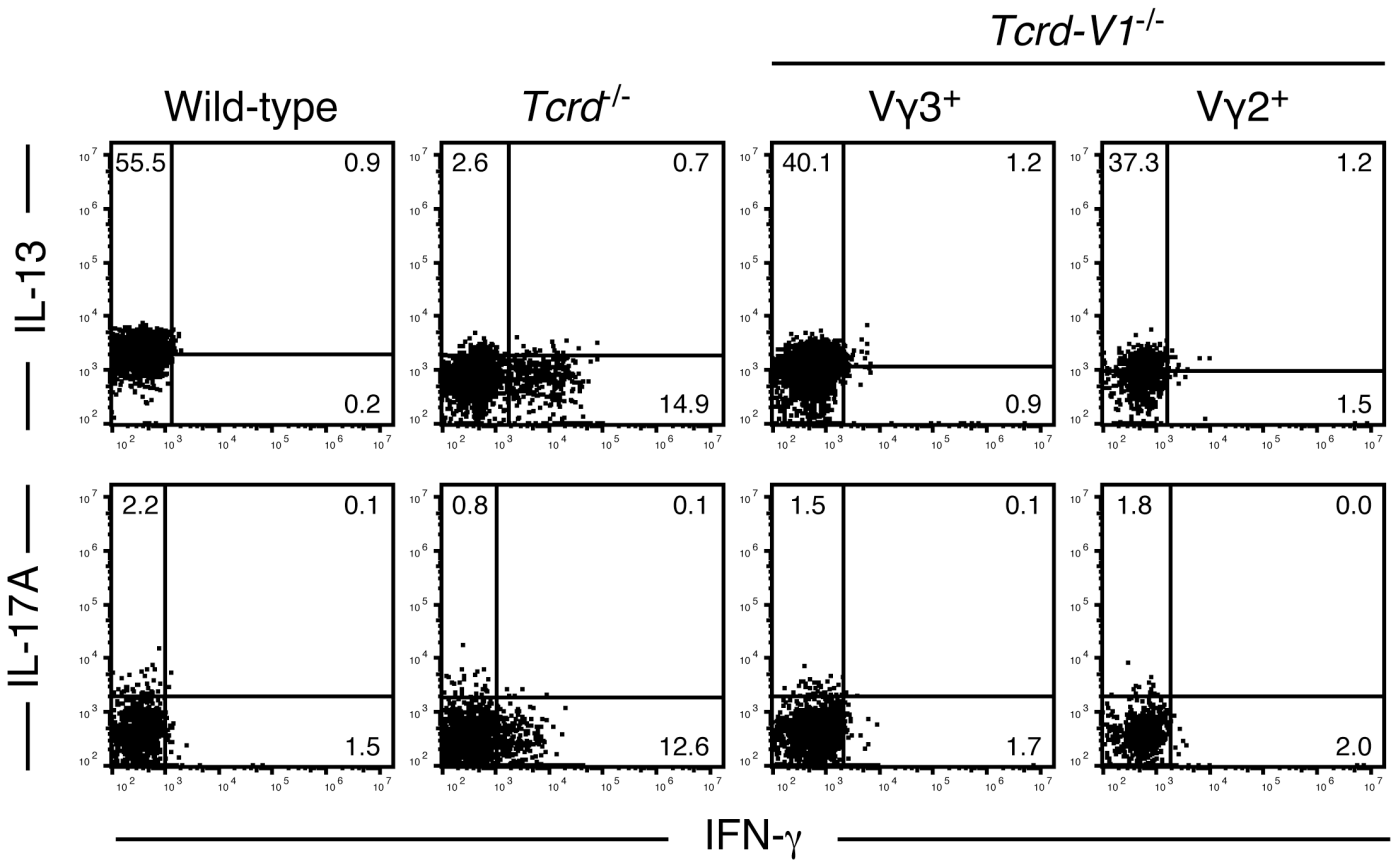
Epidermal T cells expressing TCRs that recognize the self-ligand on epidermal keratinocytes predominantly produce IL-13. Epidermal cells from the indicated adult mice were stimulated with PMA/ionomycin for 4 hours. Vγ3^+^ T cells (wild-type mice), Cβ^+^ T cells (*Tcrd* ^-/-^ mice), and Vγ3^+^ or Vγ2^+^ T cells (*Tcrd-V1* ^-/-^ mice) were gated, and intracellular IFN-γ, IL-13, and IL-17A production was analyzed by flow cytometry. Quadrant settings were determined by staining with isotype control mAbs. The numbers denote the percentages of cells in the respective quadrants. Each profile is representative of two to four independent experiments with a single mouse per experiment.

Epidermal αβ T cells of adult *Tcrd* ^-/-^ mice produced IFN-γ but not IL-13 or IL-17A upon PMA/ionomycin stimulation for 4 hours (**Figure 4**). In contrast, both epidermal Vγ3^+^ and Vγ2^+^ T cells of adult *Tcrd-V1* ^-/-^ mice, the latter of which are biased to produce IL-17A in the dermis of wild-type mice (38, 51, 52), predominantly produced IL-13 but not IFN-γ or IL-17A (**Figure 4**). These results suggest that ligand-dependent TCR signaling in the epidermis is needed for the induction and/or maintenance of the rapid IL-13-producing capacity of epidermal T cells.

### TCR signaling through the p38 MAPK pathway promotes the differentiation of neonatal DETCs into IL-13-producing cells, whereas mammalian target of rapamycin complex 1 (mTORC1) signaling suppresses differentiated IL-13-producing cells

**Figure 5:**
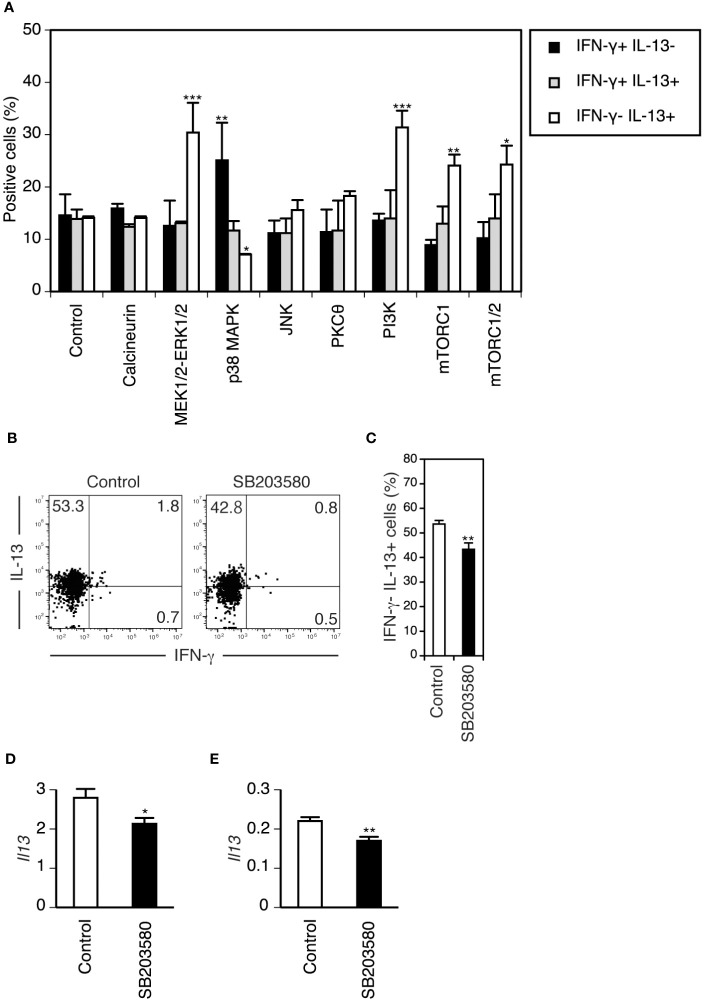
TCR signaling through the p38 MAPK pathway promotes the differentiation of neonatal DETCs into IL-13-producing cells *in vitro* and the maintenance of the rapid IL-13-producing capacity of adult DETCs *in vivo*, whereas mTORC1 signaling suppresses differentiated IL-13-producing cells *in vitro*. **(A)** Day 1 neonatal epidermal cells were stimulated with immobilized anti-TCR mAb in the presence of DMSO (control) or inhibitors of the indicated TCR signaling pathways for 5 days and rested for 2 days. After PMA/ionomycin stimulation for 4 hours, cytokine production by gated Vγ3^+^ T cells was analyzed by flow cytometry. Representative data from three independent experiments are shown as the mean and SEM (n = 3). Significant differences compared with the control are denoted with asterisks (*P < 0.05, **P < 0.01, ***P < 0.001). **(B–E)** PBS (control) and the p38 MAPK inhibitor SB203580 were injected intradermally into each ear of an adult mouse. **(B–D)** Epidermal cells were isolated from each ear 24 hours after the injection and stimulated with PMA/ionomycin for 4 hours. **(B)** Cytokine production by gated Vγ3^+^ T cells was analyzed by flow cytometry. Quadrant settings were determined by staining with isotype control mAbs. The numbers denote the percentages of cells in the respective quadrants. Representative profiles from three independent experiments are shown. **(C)** Quantification of **(B)**. Data are expressed as the mean and SEM (n = 3). Significant difference compared with the control is denoted with asterisks (**P < 0.01). **(D)** IL-13 mRNA expression in epidermal cells after PMA/ionomycin stimulation was analyzed in triplicate by real-time RT–PCR. Representative data from two independent experiments are shown as the mean and SD. Significant difference compared with the control is denoted with an asterisk (*P < 0.05). **(E)** IL-13 mRNA expression in epidermal cells isolated 4 hours after tape-stripping *in vivo* was analyzed in triplicate by real-time RT–PCR. Representative data from two independent experiments are shown as the mean and SD. Significant difference compared with the control is denoted with asterisks (**P < 0.01).

To determine the role of TCR signaling in the functional differentiation of DETCs more directly, we used an *in vitro* differentiation model of neonatal DETCs. Neonatal epidermal cells were cultured under TCR stimulation in the presence of various inhibitors of signaling pathways downstream of TCR, and cytokine production by Vγ3^+^ T cells was analyzed after restimulation with PMA/ionomycin for 4 hours. Among the various inhibitors, only the p38 MAPK inhibitor SB203580 blocked the differentiation of IL-13-producing cells from IFN-γ-producing cells (**Figure 5A**). Therefore, it was found that TCR signaling through the p38 MAPK pathway promotes the functional switch of DETCs.

Interestingly, the addition of MEK1/2-ERK1/2, PI3K, mTORC1, and mTORC1/2 inhibitors increased IL-13-producing cells with minimal impact on IFN-γ-producing cells (**Figure 5A**). As both ERK and PI3K activate mTORC1 (53), mTORC1 activation might suppress differentiated IL-13-producing cells.

### The p38 MAPK inhibitor SB203580 blocks the maintenance of the rapid IL-13-producing capacity of adult DETCs *in vivo*

In the adult epidermis, TCRs on DETCs are triggered at steady state (30). Therefore, TCR-p38 MAPK signaling may also play a role in the maintenance of the IL-13-producing capacity of DETCs in the adult epidermis. Intradermal administration of the p38 MAPK inhibitor SB203580 24 hours before analysis decreased DETCs with the IL-13-producing capacity in the adult epidermis (**Figures 5B, C**). IL-13 mRNA expression in epidermal cells upon short-term stimulation with PMA/ionomycin *in vitro* (**Figure 5D**) and in response to tape-stripping *in vivo* (**Figure 5E**) was suppressed by SB203580 pretreatment. As DETCs are the only cells in the epidermis that produce IL-13 (22), these results suggest that continuous TCR signaling through the p38 MAPK pathway is also needed for maintaining the rapid IL-13-producing capacity of adult DETCs under steady-state conditions.

### The metabolic switch in DETCs from high glycolytic capacity to higher mitochondrial dependence occurs between 2 and 4 weeks after birth

A recent study showed that IFN-γ-producing γδ T cells use glycolysis for proliferation and to maintain effector functions, but IL-17A-producing γδ T cells are dependent on mitochondrial oxidative phosphorylation and fatty acid oxidation (54). As mTORC1, which regulates cellular metabolism, suppressed IL-13-producing cells in our *in vitro* differentiation model (**Figure 5A**), the functional switch of DETCs from IFN-γ-producing cells to IL-13-producing cells may be associated with the metabolic switch from mTORC1-dependent metabolic pathways to those less dependent on mTORC1.

**Figure 6:**
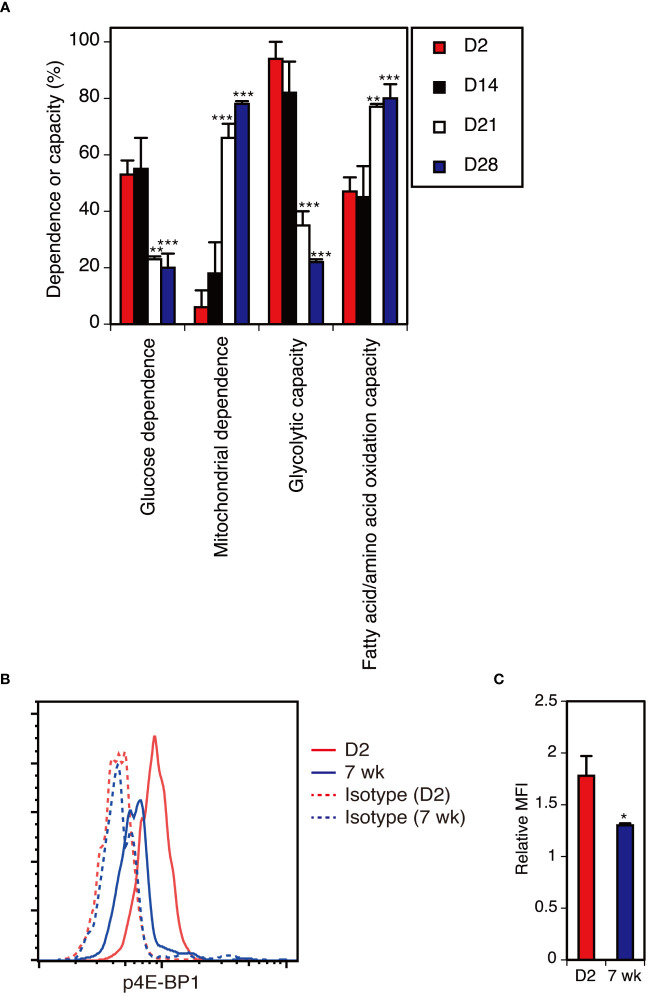
The metabolic switch in DETCs from high glycolytic capacity to higher mitochondrial dependence occurs between 2 and 4 weeks after birth. **(A)** Epidermal cells isolated at the indicated time points were analyzed without stimulation for the metabolic dependence or capacity of gated Vγ3^+^ T cells by SCENITH™. Representative data from two independent experiments are shown. In each experiment, cells pooled from 3-7 mice were analyzed in triplicate. Data are expressed as the mean and SD. Significant differences compared with D2 are denoted with asterisks (**P < 0.01, ***P < 0.001). **(B)** Epidermal cells were isolated from D2 neonatal mice and 7-week-old adult mice and analyzed without stimulation for intracellular p4E-BP1 expression in gated Vγ3^+^ T cells. Representative profiles from two independent experiments are shown. In each experiment, cells pooled from 3-4 neonatal mice and a single adult mouse were analyzed in triplicate. **(C)** Quantification of **(B)**. Relative MFI was determined as (geometric MFI of p4E-BP1)/(geometric MFI of isotype control). Data are expressed as the mean and SD. Significant difference compared with D2 is denoted with an asterisk (*P < 0.05).

To analyze the metabolic profiles of DETCs at different ages, we used the recently developed flow cytometry-based method SCENITH™ (55). D2 neonatal and D14 DETCs displayed higher glucose dependence and higher glycolytic capacity than D21 and D28 adult DETCs (**Figure 6A**). By D21, DETCs showed low glucose dependence and low glycolytic capacity, with a subsequent high dependence on mitochondrial oxidative phosphorylation and fatty acid/amino acid oxidation (**Figure 6A**). This metabolic reprogramming took place between 2 and 4 weeks after birth (**Figure 6A**) and thus occurred later than the functional switch observed during the perinatal period (**Figure 3**). As DETCs have been shown to cease massive proliferation in the epidermis by 2 weeks after birth in C57BL/6 mice (37), mTORC1 activity may be attenuated at this time and maintained at low levels in adult DETCs under steady-state conditions.

### Attenuated mTORC1 activity in adult DETCs under steady-state conditions

To determine whether mTORC1 activity is diminished in adult DETCs under steady-state conditions, we compared the levels of phosphorylated 4E-BP1 (p4E-BP1), an mTORC1 downstream target, between neonatal and adult DETCs. Compared with D2 neonatal DETCs, p4E-BP1 levels were lower in adult DETCs (**Figures 6B, C**). Therefore, mTORC1 activity was found to be attenuated in adult DETCs under steady-state conditions.

### Glycolysis inhibition decreases IFN-γ-producing cells but not IL-13-producing cells in adult DETCs during the late phase of activation

**Figure 7:**
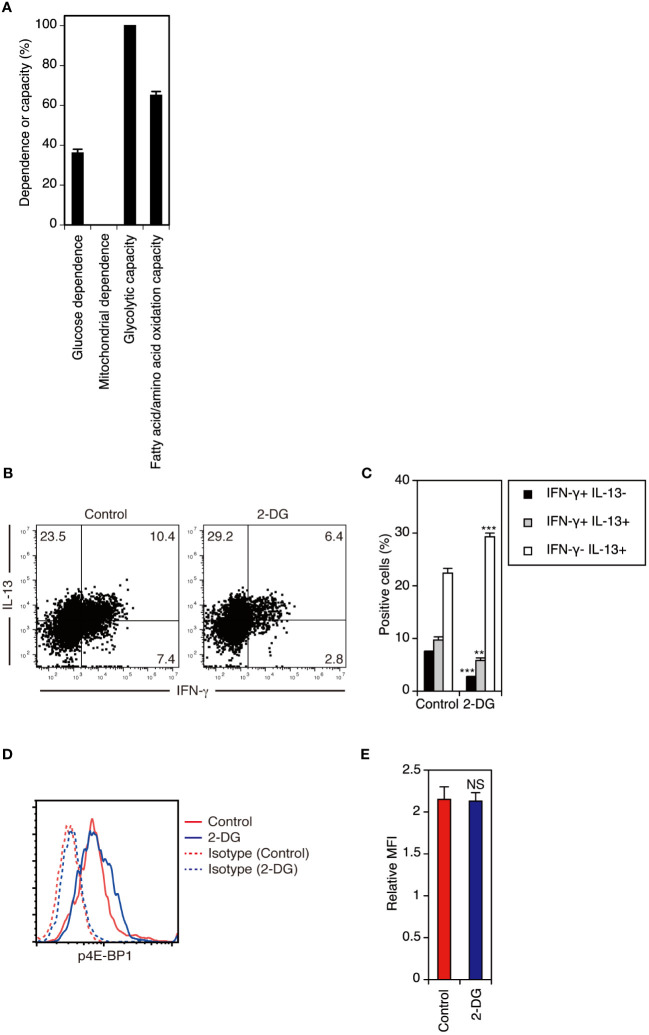
Inhibition of glycolysis decreases IFN-γ-producing cells but not IL-13-producing cells in adult DETCs during the late phase of activation. **(A)** Adult epidermal cells stimulated with anti-TCR mAb for 3 days *in vitro* were analyzed for the metabolic dependence or capacity of gated Vγ3^+^ T cells by SCENITH™. Representative data from two independent experiments are shown. In each experiment, cells pooled from 2 mice were analyzed in triplicate. Data are expressed as the mean and SD. **(B)** Adult epidermal cells were stimulated with anti-TCR mAb in the presence of DMSO (control) or 2-DG for 3 days *in vitro*. Vγ3^+^ T cells were gated, and intracellular IFN-γ and IL-13 production was analyzed by flow cytometry. Quadrant settings were determined by staining with isotype control mAbs. The numbers denote the percentages of cells in the respective quadrants. Representative profiles from two independent experiments are shown. In each experiment, cells pooled from 2 mice were analyzed in triplicate. **(C)** Quantification of **(B)**. Data are expressed as the mean and SD. Significant differences compared with the control are denoted with asterisks (**P < 0.01, ***P < 0.001). **(D)** Adult epidermal cells stimulated with anti-TCR mAb in the presence of DMSO (control) or 2-DG for 3 days *in vitro* were analyzed for intracellular p4E-BP1 expression in gated Vγ3^+^ T cells. Representative profiles from two independent experiments are shown. In each experiment, cells pooled from 2 mice were analyzed in triplicate. **(E)** Quantification of **(D)**. Relative MFI was determined as (geometric MFI of p4E-BP1)/(geometric MFI of isotype control). Data are expressed as the mean and SD. Compared with the control, 2-DG treatment did not diminish p4E-BP1 expression in stimulated DETCs (NS, not significant).

Finally, we determined whether cellular metabolism regulates cytokine production by adult DETCs. In contrast to adult DETCs under steady-state conditions (**Figure 6A**), adult DETCs stimulated with anti-TCR mAb for 3 days *in vitro* showed high glycolytic capacity (**Figure 7A**). Although adult DETCs predominantly produce IL-13 during the early phase of activation, adult DETCs stimulated for 3 days *in vitro* produced both IL-13 and IFN-γ (**Figures 7B, C**). Inhibition of glycolysis with 2-deoxy-D-glucose (2-DG) decreased IFN-γ-producing cells but not IL-13-producing cells in adult DETCs stimulated with anti-TCR mAb for 3 days *in vitro* (**Figures 7B, C**). As TCR-stimulated DETCs cultured in the presence or absence of 2-DG had equivalent levels of p4E-BP1 (**Figures 7D, E**), inhibition of glycolysis did not alter mTORC1 activity in adult DETCs. Therefore, it was found that glycolysis acts downstream of mTORC1 in the regulation of DETC cytokine production.

## Discussion

We showed that preprogrammed IFN-γ-producing Vγ3^+^ T cells differentiate into rapid IL-13 producers in the perinatal epidermis. This functional switch was promoted by TCR signaling through the p38 MAPK pathway. The downstream substrate of p38 MAPK in perinatal DETCs is currently unknown, but a likely candidate is GATA3 because phosphorylation of GATA3 by p38 MAPK is crucial for GATA3 nuclear translocation and IL-13 production in type 2 helper T cells (56) and group 2 innate lymphoid cells (57). As TCR signaling activates p38 MAPK not only through the canonical MAPK cascade but also through direct phosphorylation of p38 MAPK on a non-canonical activating residue by LCK-ZAP70 (58), it is also important to identify the upstream signaling pathway of p38 MAPK in perinatal DETCs in future studies.

Factors present in the perinatal epidermal microenvironment other than TCR signaling may also contribute to the induction of the IL-13-producing capacity of DETCs. Thus far, we have not yet identified such a factor. Treatment of E17 fetal thymocytes with IL-2, IL-4, IL-7, IL-15, TGF-β, or ATP did not induce the IL-13-producing capacity of Vγ3^+^ T cells (unpublished data). We also confirmed that adult DETCs of both TSLP receptor-deficient mice and wild-type mice treated with anti-IL-25 and anti-IL-33 mAbs *in vivo* produced IL-13 but not IFN-γ upon short-term PMA/ionomycin stimulation (unpublished data). Nevertheless, the involvement of cognate signaling through interactions between DETCs and epidermal keratinocytes has not been addressed and warrants further investigation.

We demonstrated that signaling through the p38 MAPK pathway was also needed for the maintenance of the rapid IL-13-producing capacity of adult DETCs under steady-state conditions. Although p38 MAPK activation by a receptor other than TCR could be responsible for this finding, our data are consistent with those of a recent study showing that chronic intradermal administration of anti-Skint1 mAb resulted in the loss of IL-13 expression by adult DETCs (59) and together support the notion that constitutive TCR signaling at steady state through Skint1-dependent recognition of the self-ligand expressed on healthy keratinocytes (‘normality sensing’) maintains DETCs in a poised state to rapidly respond to epidermal stress (59). DETCs primarily produce IL-13 when activated *in vivo* after exposure to a variety of environmental stressors (22) and even after acute upregulation of transgenic NKG2D ligands on epidermal keratinocytes (24). Therefore, IL-13 production can be triggered in steady-state DETCs not only by TCR signaling via stress-induced upregulation of the TCR ligand but also by signaling through other stress-sensing receptors, including NKG2D, to maintain epidermal homeostasis (22).

We identified a role of cellular metabolism in the regulation of cytokine production by adult DETCs. Proliferating neonatal DETCs had high glycolytic capacity, whereas adult DETCs at steady state were more dependent on mitochondrial metabolism. This metabolic switch occurred between 2 and 4 weeks after birth. This is consistent with the fact that DETCs cease massive postnatal proliferation at this time in C57BL/6 mice (37). Accordingly, mTORC1 activity in adult DETCs under steady-state conditions was attenuated compared with that in proliferating neonatal DETCs. Inhibition of glycolysis decreased adult DETCs producing IFN-γ during the late phase of activation. As the inhibition of glycolysis resulted in a relative increase in the frequency of IL-13-producing DETCs, IL-13-producing DETCs redifferentiated into IFN-γ-producing cells during the late phase of activation. The (re)acquisition of the IFN-γ-producing capacity would require higher energy fueled by mTORC1-dependent glycolysis through sustained signaling than IL-13 production. Conversely, the low glycolytic capacity of adult DETCs at steady state contributes to the predominant production of IL-13 over IFN-γ early after activation as the former is less dependent on glycolysis than the latter.

mTORC1 is needed for the proliferation and survival of peripheral γδ T cells and is essential for the differentiation of both IFN-γ-producing and IL-17A-producing γδ T cells (60). *In vitro* treatment of adult DETCs with a high dose (20 ng/mL) of rapamycin inhibits their proliferation and induces autophagy (61). Therefore, a basal level of mTORC1 activity through low levels of TCR signaling and/or IL-15 receptor signaling is essential for the maintenance of DETCs at steady state. Full activation of DETCs and IFN-γ production would require enhanced mTORC1 activity and high glycolytic capacity to meet increased metabolic needs. To avoid complete blocking of mTORC1 activity, we used a low dose (5 ng/mL) of rapamycin for inhibiting mTORC1 activation in our *in vitro* neonatal DETC differentiation model. Although low-dose rapamycin treatment was reported to activate mTORC2 (62), the involvement of mTORC2 in the increase in IL-13-producing cells by rapamycin treatment was unlikely in our experiments because the addition of Torin 1, which inhibits both mTORC1 and mTORC2, also increased IL-13-producing cells. In our *in vitro* differentiation model, inhibition of mTORC1-dependent glycolysis might prevent differentiated IL-13-producing cells from redifferentiating into IFN-γ-producing cells and result in an increase in IL-13-producing cells.

In summary, we demonstrated that TCR signaling through the p38 MAPK pathway promotes the differentiation of IL-13-producing Vγ3^+^ T cells in the perinatal epidermis and that constitutive TCR signaling at steady state is also needed for maintaining the rapid IL-13-producing capacity of adult DETCs. In addition, the low mTORC1 activity and low glycolytic capacity of adult DETCs at steady state also regulate the rapid IL-13 response and delayed IFN-γ production after activation. A variety of sometimes conflicting effector functions of DETCs have been identified (7, 20–25). The effector functions of DETCs may be fine-tuned by their metabolic states depending on the epidermal microenvironment. Whether the functions of DETCs other than cytokine production (e.g., growth factor production, cytotoxicity) are regulated by cellular metabolism remains to be determined in future studies.

## Materials and methods

### Mice

C57BL/6J mice were purchased from Japan SLC (Hamamatsu, Japan). *Tcrd* ^-/-^ mice (63) were purchased from the Jackson Laboratory (Bar Harbor, ME). *Tcrd-V1* ^-/-^ mice (50) were a gift from Yasunobu Yoshikai (Division of Host Defense, Medical Institute of Bioregulation, Kyusyu University, Fukuoka, Japan). *TSLP-R* ^-/-^ mice (64) were a gift from Steven F. Ziegler (Benaroya Research Institute, Seattle, WA). All mice were bred and maintained on a C57BL/6 background in the animal facility of Kagoshima University under specific pathogen-free conditions. Female mice at 4-12 weeks of age were used as adult mice. Fetuses and postnatal mice younger than 4 weeks were used irrespective of sex. Fetuses were obtained from timed pregnant mice. The plug date was defined as embryonic day 0 (E0).

### Cells

To isolate epidermal cells, the skin was floated dermal-side down on 1% trypsin (Gibco, Waltham, MA) in PBS for 30 minutes at 37°C. The epidermis was separated and collected in Iscove’s modified Dulbecco’s medium (IMDM, Gibco) supplemented with 10% FCS (Sigma–Aldrich, St. Louis, MO) and 0.025% DNase I (Sigma–Aldrich). Single-cell suspensions were obtained by mechanical agitation and sequential filtration through 70- and 30-μm nylon meshes.

DETCs were purified from epidermal cells by positive magnetic selection using an anti-integrin β7 mAb because DETCs are the only cells in the normal epidermis that express the integrin β7 chain (65). After preincubation with anti-CD16/CD32 mAb (clone 2.4G2; BD Biosciences, Franklin Lakes, NJ), cells were stained with PE-conjugated anti-integrin β7 mAb (clone M293, BD Biosciences), followed by incubation with magnetic particles conjugated with anti-PE mAb (BD IMag™ Anti-R-PE Magnetic Particles-DM, BD Biosciences). The labeled cells were isolated using the BD IMag™ Cell Separation Magnet (BD Biosciences) according to the manufacturer’s instructions.

To isolate dermal cells, the skin was floated dermal-side down on 1.2 U/mL dispase II (Roche Diagnostics, Basel, Switzerland) in IMDM for 30 minutes at 37°C. After removing the epidermis, small pieces of the dermis were digested in IMDM containing 0.01% DNase I and 250 U/mL collagenase IV (Sigma–Aldrich) for 30 minutes in a shaking water bath at 37°C. The digested dermis was filtered through 70- and 30-μm nylon meshes.

Fetal thymocytes were obtained by teasing the thymic lobes with fine forceps and filtering through a 70-μm nylon mesh.

Blood was collected in 20 mM EDTA in PBS. Lymphocytes were isolated by density gradient centrifugation on Lympholyte™-M Cell Separation Media (Cedarlane Laboratories, Burlington, Canada) for 15 minutes at 1000 × g.

### Flow cytometry

Cells were resuspended in PBS supplemented with 2% FCS and 0.1% NaN_3_. After preincubation with anti-CD16/CD32 mAb, cells were stained with the following mAbs: FITC-, PE-, BD Horizon™ BB700-, or biotin-conjugated anti-TCR Vγ3 (clone 536, BD Biosciences or BioLegend, San Diego, CA), FITC-conjugated anti-CD3 (clone 145-2C11, eBioscience, Waltham, MA), FITC-conjugated anti-IA^b^ (clone AF6-120.1, BD Biosciences), biotin-conjugated anti-TCR Cβ (clone H57-597, BD Biosciences), biotin-conjugated anti-CD27 (clone LG.7F9, eBioscience), BB700-conjugated anti-TCR Vγ2 (clone UC3-10A6, BD Biosciences), and FITC-, PE-, BB700-, or biotin-conjugated isotype control mAbs (BD Biosciences or eBioscience). Biotin-conjugated mAbs were visualized with FITC- or PE-Cy5™-conjugated streptavidin (SouthernBiotech, Birmingham, AL or BD Biosciences).

For intracellular cytokine staining, cells were stimulated with 25 ng/mL PMA (Sigma–Aldrich) and 1 μg/mL ionomycin (Sigma–Aldrich) in the presence of brefeldin A (GolgiPlug™, BD Biosciences) for 4 hours at 37°C. TCR-stimulated cells were incubated with brefeldin A for the last 4 hours. After surface staining, the cells were fixed and permeabilized using Cytofix/Cytoperm™ (BD Biosciences) for 20 minutes at 4°C. Cells were washed and stained in Perm/Wash™ buffer (BD Biosciences) with Alexa Fluor™ 488-conjugated anti-IFN-γ (clone XMG1.2, BD Biosciences), PE-conjugated anti-IL-13 (clone eBio13A, eBioscience), PE-conjugated anti-IL-17A (clone TC11-18H10, BD Biosciences), and Alexa Fluor™ 488- or PE-conjugated isotype control mAbs (BD Biosciences or eBioscience).

For intracellular p4E-BP1 staining, after surface staining, cells were fixed and permeabilized using Cytofix/Cytoperm™ and washed and stained in Perm/Wash™ buffer with Alexa Fluor™ 488-conjugated anti-p4E-BP1 (Thr37/46) mAb (clone 236B4, Cell Signaling Technology, Danvers, MA) or Alexa Fluor™ 488-conjugated isotype control mAb (Cell Signaling Technology).

After gating on forward and side scatters and viable cells as previously described (29, 65), cells were analyzed on a CytoFLEX flow cytometer with CytExpert software (Beckman Coulter, Brea, CA), and the data were analyzed using FlowJo™ software (Tree Star, Ashland, OR).

### TCR stimulation

Purified DETCs were stimulated on 96-well plates (5 × 10^4^ cells/well) coated with 10 μg/mL anti-TCR Cδ mAb (UC7-13D5, BD Biosciences) or isotype control mAb (BD Biosciences) in IMDM supplemented with 10% FCS and 50 μM 2-mercaptoethanol (Nacalai Tesque, Kyoto, Japan) for 24 hours at 37°C. Culture supernatants were harvested and replaced with fresh culture medium, and DETCs were stimulated for an additional 24 hours. Cytokine levels in the supernatants were determined using Quantikine™ ELISA kits (R&D Systems, Minneapolis, MN).

Epidermal cells were stimulated on 24-well plates (1 × 10^6^ cells/well) coated with 10 μg/mL anti-TCR Cδ mAb in IMDM supplemented with 10% FCS, 50 μM 2-mercaptoethanol, and 10 ng/mL recombinant mouse IL-2 (R&D Systems) in the presence of DMSO (Sigma–Aldrich) or 3 mM 2-DG (MedChemExpress, Monmouth Junction, NJ) for 3 days at 37°C. At the time of use, DETCs were harvested by incubation with 1 mM EDTA in PBS for 3 minutes.

### Real-time RT–PCR

Total RNA was extracted from the cells using the RNeasy™ Plus Mini kit (Qiagen, Venlo, Netherlands) and reverse transcribed using the SuperScript™ III First-Strand Synthesis System for RT–PCR (Invitrogen, Waltham, MA) with random hexamers. The cDNA was subjected to quantitative real-time PCR in triplicate using Thermal Cycler Dice™ Real Time System (Takara, Kusatsu, Japan) with FastStart Universal SYBR™ Green Master (Roche Diagnostics). All primers were purchased from Takara. Primer sequences are available upon request. The cycling conditions were 95°C for 10 minutes, followed by 40 cycles of 95°C for 15 seconds and 60°C for 1 minute. Threshold cycle (Ct) values were determined, and mRNA expression relative to that of the *Actβ* mRNA was calculated as 2^-ΔCt^.

### ATP treatment of fetal thymocytes

E17 fetal thymocytes were cultured on 24-well plates (2 × 10^6^ cells/well) in IMDM supplemented with 10% FCS and 50 μM 2-mercaptoethanol in the presence of PBS or 3 mM ATP (Sigma–Aldrich) for 30 minutes at 37°C.

### *In vitro* differentiation model of neonatal DETCs

Epidermal cells isolated from day 1 neonatal mice were stimulated on 24-well plates (1 × 10^6^ cells/well) coated with 10 μg/mL anti-TCR Cδ mAb in IMDM supplemented with 10% FCS, 50 μM 2-mercaptoethanol, and 10 ng/mL recombinant mouse IL-2 for 5 days at 37°C. Various inhibitors of signaling pathways downstream of TCR were added during this period. Cells were harvested and rested on uncoated plates in the same culture medium without inhibitors for 2 days at 37°C to allow the recovery of TCR expression before restimulation with PMA/ionomycin followed by intracellular cytokine staining.

Optimal concentrations of the inhibitors were predetermined as the maximum concentrations that did not affect viable Vγ3^+^ T-cell yields after the cultures. The following inhibitors were used at the indicated concentrations: calcineurin inhibitor cyclosporin A (0.01 μM; Cell Signaling Technology), MEK1/2-ERK1/2 inhibitor U0126 (5 μM, Cell Signaling Technology), p38 MAPK inhibitor SB203580 (10 μM, Cell Signaling Technology), JNK inhibitor SP600125 (5 μM, Cell Signaling Technology), PKCθ inhibitor sotrastaurin (0.1 μM; Abcam, Cambridge, UK), PI3K inhibitor LY294002 (5 μM, Cell Signaling Technology), mTORC1 inhibitor rapamycin (5 ng/mL, Sigma–Aldrich), and mTORC1/2 inhibitor Torin 1 (0.05 μM, Cell Signaling Technology).

### *In vivo* treatment with the p38 MAPK inhibitor and tape-stripping

PBS or 10 μg SB203580 in 25 μL PBS was administered by intradermal injections into the dorsal and ventral sides of the ear pinna using a 29-gauge needle under inhalation anesthesia. Epidermal cells were isolated 24 hours after the injection and stimulated for 4 hours with PMA/ionomycin. To activate DETCs *in situ* by mild tissue abrasion induced by tape-stripping, the stratum corneum was removed from both sides of the earlobe by application and removal of cellophane tape (Scotch™, 3M, St. Paul, MN) seven times (22, 24) 24 hours after the injection. Epidermal cells were isolated 4 hours after tape stripping.

### Measurement of metabolic dependence and capacity

SCENITH™ was performed as previously described (55) using the SCENITH™ kit containing all reagents and protocols (obtained from www.scenith.com/try-it). Briefly, cells were treated on 24-well plates (1 × 10^6^ cells/well) with DMSO (control), 100 mM 2-DG, 1 μM oligomycin, or a combination of 2-DG and oligomycin for 40 minutes (for resting cells) or 30 minutes (for activated cells) at 37°C. Puromycin (10 μg/mL) was added for 40 minutes (for resting cells) or for the last 15 minutes (for activated cells) at 37°C. After surface staining, the cells were fixed and permeabilized using Cytofix/Cytoperm™ and washed and stained in Perm/Wash™ buffer with Alexa Fluor 488-conjugated anti-puromycin mAb (clone R4743L-E8) for 30 minutes at 4°C. The impact of the various metabolic inhibitors was quantified as previously described (55).

### Statistical analysis

Differences between the two groups were evaluated by *t*-test. Dunnett’s test was used for multiple comparisons to a control. All reported *P* values are two-tailed, with a *P* value < 0.05 considered significant. Statistical calculations were performed using JMP™ software (SAS Institute, Cary, NC).

## Data availability statement

The raw data supporting the conclusions of this article will be made available by the authors without undue reservation.

## Ethics statement

The animal study was approved by the Animal Care Committee of Kagoshima University. The study was conducted in accordance with the local legislation and institutional requirements.

## Author contributions

AI: Conceptualization, Investigation, Validation, Writing – original draft, Visualization. KK: Conceptualization, Funding acquisition, Investigation, Validation, Writing – original draft, Writing – review & editing, Formal analysis, Visualization. AN-T: Investigation, Validation, Writing – review & editing. RA: Funding acquisition, Writing – review & editing, Methodology, Resources. TK: Supervision, Writing – review & editing.

## References

[1] Allison JP Havran WL. The immunobiology of T cells with invariant γδ antigen receptors. Annu Rev Immunol. (1991) 9:679–705. doi: 10.1146/annurev.iy.09.040191.003335 1832874

[2] Hayday AC. γδ cells: A right time and a right place for a conserved third way of protection. Annu Rev Immunol. (2000) 18:975–1026. doi: 10.1146/annurev.immunol.18.1.975 10837080

[3] Carding SR Egan PJ. γδ T cells: Functional plasticity and heterogeneity. Nat Rev Immunol. (2002) 2:336–45. doi: 10.1038/nri797 12033739

[4] Hayday AC. γδ T cells and the lymphoid stress-surveillance response. Immunity. (2009) 31:184–96. doi: 10.1016/j.immuni.2009.08.006 19699170

[5] Bonneville M O'Brien RL Born WK. γδ T cell effector functions: a blend of innate programming and acquired plasticity. Nat Rev Immunol. (2010) 10:467–78. doi: 10.1038/nri2781 20539306

[6] Vantourout P Hayday A. Six-of-the-best: Unique contributions of γδ T cells to immunology. Nat Rev Immunol. (2013) 13:88–100. doi: 10.1038/nri3384 23348415 PMC3951794

[7] Nielsen MM Witherden DA Havran WL. γδ T cells in homeostasis and host defence of epithelial barrier tissues. Nat Rev Immunol. (2017) 17:733–45. doi: 10.1038/nri.2017.101 PMC5771804 28920588

[8] Ribot JC Lopes N Silva-Santos B. γδ T cells in tissue physiology and surveillance. Nat Rev Immunol. (2021) 21:221–32. doi: 10.1038/s41577-020-00452-4 33057185

[9] Zhu J Yamane H Paul WE. Differentiation of effector CD4 T cell populations. Annu Rev Immunol. (2010) 28:445–89. doi: 10.1146/annurev-immunol-030409-101212 PMC3502616 20192806

[10] Muñoz-Ruiz M Sumaria N Pennington DJ Silva-Santos B. Thymic determinants of γδ T cell differentiation. Trends Immunol. (2017) 38:336–44. doi: 10.1016/j.it.2017.01.007 28285814

[11] Sumaria N Martin S Pennington DJ. Developmental origins of murine γδ T-cell subsets. Immunology. (2019) 156:299–304. doi: 10.1111/imm.13032 30552818 PMC6418442

[12] Parker ME Ciofani M. Regulation of γδ T cell effector diversification in the thymus. Front Immunol. (2020) 11:42. doi: 10.3389/fimmu.2020.00042 32038664 PMC6992645

[13] Jensen KDC Su X Shin S Li L Youssef S Yamasaki S. Thymic selection determines γδ T cell effector fate: antigen-naive cells make interleukin-17 and antigen-experienced cells make interferon γ. Immunity. (2008) 29:90–100. doi: 10.1016/j.immuni.2008.04.022 18585064 PMC2601709

[14] Ribot JC deBarros A Pang DJ Neves JF Peperzak V Roberts SJ. CD27 is a thymic determinant of the balance between interferon-γ- and interleukin 17-producing γδ T cell subsets. Nat Immunol. (2009) 10:427–36. doi: 10.1038/ni.1717 PMC4167721 19270712

[15] Turchinovich G Hayday AC. Skint-1 identifies a common molecular mechanism for the development of interferon-γ-secreting versus interleukin-17-secreting γδ T cells. Immunity. (2011) 35:59–68. doi: 10.1016/j.immuni.2011.04.018 21737317

[16] Muñoz-Ruiz M Ribot JC Grosso AR Goncalves-Sousa N Pamplona A Pennington DJ. TCR signal strength controls thymic differentiation of discrete proinflammatory γδ T cell subsets. Nat Immunol. (2016) 17:721–7. doi: 10.1038/ni.3424 PMC4875770 27043412

[17] Sumaria N Grandjean CL Silva-Santos B Pennington DJ. Strong TCRγδ signaling prohibits thymic development of IL-17A-secreting γδ T cells. Cell Rep. (2017) 19:2469–76. doi: 10.1016/j.celrep.2017.05.071 PMC5489697 28636936

[18] Zuberbuehler MK Parker ME Wheaton JD Espinosa JR Salzler HR Park E. The transcription factor c-Maf is essential for the commitment of IL-17-producing γδ T cells. Nat Immunol. (2019) 20:73–85. doi: 10.1038/s41590-018-0274-0 30538336 PMC6294311

[19] Garman RD Doherty PJ Raulet DH. Diversity, rearrangement, and expression of murine T cell gamma genes. Cell. (1986) 45:733–42. doi: 10.1016/0092-8674(86)90787-7 3486721

[20] Thelen F Witherden DA. Get in touch with dendritic epithelial T cells! Front Immunol. (2020) 11:1656. doi: 10.3389/fimmu.2020.01656 32849572 PMC7403176

[21] Sharp LL Jameson JM Cauvi G Havran WL. Dendritic epidermal T cells regulate skin homeostasis through local production of insulin-like growth factor 1. Nat Immunol. (2005) 6:73–9. doi: 10.1038/ni1152 15592472

[22] Dalessandri T Crawford G Hayes M Castro Seoane R Strid J. IL-13 from intraepithelial lymphocytes regulates tissue homeostasis and protects against carcinogenesis in the skin. Nat Commun. (2016) 7:12080. doi: 10.1038/ncomms12080 27357235 PMC4931319

[23] Jameson J Ugarte K Chen N Yachi P Fuchs E Boismenu R. A role for skin γδ T cells in wound repair. Science. (2002) 296:747–9. doi: 10.1126/science.1069639 11976459

[24] Strid J Sobolev O Zafirova B Polic B Hayday A. The intraepithelial T cell response to NKG2D-ligands links lymphoid stress surveillance to atopy. Science. (2011) 334:1293–7. doi: 10.1126/science.1211250 PMC3842529 22144628

[25] Girardi M Oppenheim DE Steele CR Lewis JM Glusac E Filler R. Regulation of cutaneous malignancy by γδ T cells. Science. (2001) 294:605–9. doi: 10.1126/science.1063916 11567106

[26] Havran WL Chien YH Allison JP. Recognition of self antigens by skin-derived T cells with invariant γδ antigen receptors. Science. (1991) 252:1430–2. doi: 10.1126/science.1828619 1828619

[27] Jameson JM Cauvi G Witherden DA Havran WL. A keratinocyte-responsive γδ TCR is necessary for dendritic epidermal T cell activation by damaged keratinocytes and maintenance in the epidermis. J Immunol. (2004) 172:3573–9. doi: 10.4049/jimmunol.172.6.3573 15004158

[28] Komori HK Witherden DA Kelly R Sendaydiego K Jameson JM Teyton L. Cutting edge: dendritic epidermal γδ T cell ligands are rapidly and locally expressed by keratinocytes following cutaneous wounding. J Immunol. (2012) 188:2972–6. doi: 10.4049/jimmunol.1100887 PMC3311739 22393149

[29] Ibusuki A Kawai K Yoshida S Uchida Y Nitahara-Takeuchi A Kuroki K. NKG2D triggers cytotoxicity in murine epidermal γδ T cells via PI3K-dependent, Syk/ZAP70-independent signaling pathway. J Invest Dermatol. (2014) 134:396–404. doi: 10.1038/jid.2013.353 23962808

[30] Chodaczek G Papanna V Zal MA Zal T. Body-barrier surveillance by epidermal γδ TCRs. Nat Immunol. (2012) 13:272–82. doi: 10.1038/ni.2240 PMC3288780 22327568

[31] Boyden LM Lewis JM Barbee SD Bas A Girardi M Hayday AC. *Skint1*, the prototype of a newly identified immunoglobulin superfamily gene cluster, positively selects epidermal γδ T cells. Nat Genet. (2008) 40:656–62. doi: 10.1038/ng.108 PMC4167720 18408721

[32] Jandke A Melandri D Monin L Ushakov DS Laing AG Vantourout P. Butyrophilin-like proteins display combinatorial diversity in selecting and maintaining signature intraepithelial γδ T cell compartments. Nat Commun. (2020) 11:3769. doi: 10.1038/s41467-020-17557-y 32724083 PMC7387338

[33] Xiong N Kang C Raulet DH. Positive selection of dendritic epidermal γδ T cell precursors in the fetal thymus determines expression of skin-homing receptors. Immunity. (2004) 21:121–31. doi: 10.1016/j.immuni.2004.06.008 15345225

[34] Jin Y Xia M Sun A Saylor CM Xiong N. CCR10 is important for the development of skin-specific γδT cells by regulating their migration and location. J Immunol. (2010) 185:5723–31. doi: 10.4049/jimmunol.1001612 PMC3037513 20937851

[35] Xia M Qi Q Jin Y Wiest DL August A Xiong N. Differential roles of IL-2-inducible T cell kinase-mediated TCR signals in tissue-specific localization and maintenance of skin intraepithelial T cells. J Immunol. (2010) 184:6807–14. doi: 10.4049/jimmunol.1000453 PMC2941197 20483745

[36] Romani N Schuler G Fritsch P. Ontogeny of Ia-positive and Thy-1-positive leukocytes of murine epidermis. J Invest Dermatol. (1986) 86:129–33. doi: 10.1111/1523-1747.ep12284135 2875114

[37] Elbe A Tschachler E Steiner G Binder A Wolff K Stingl G. Maturational steps of bone marrow-derived dendritic murine epidermal cells: phenotypic and functional studies on Langerhans cells and Thy-1(+) dendritic epidermal cells in the perinatal period. J Immunol. (1989) 143:2431–8. doi: 10.4049/jimmunol.143.8.2431 2571637

[38] Cai Y Shen X Ding C Qi C Li K Li X. Pivotal role of dermal IL-17-producing γδ T cells in skin inflammation. Immunity. (2011) 35:596–610. doi: 10.1016/j.immuni.2011.08.001 21982596 PMC3205267

[39] Macleod AS Hemmers S Garijo O Chabod M Mowen K Witherden DA. Dendritic epidermal T cells regulate skin antimicrobial barrier function. J Clin Invest. (2013) 123:4364–74. doi: 10.1172/JCI70064 PMC3784546 24051381

[40] Shibata K Yamada H Nakamura R Sun X Itsumi M Yoshikai Y. Identification of CD25(+) γδ T cells as fetal thymus-derived naturally occurring IL-17 producers. J Immunol. (2008) 181:5940–7. doi: 10.4049/jimmunol.181.9.5940 18941182

[41] Sugaya M Nakamura K Tamaki K. Interleukins 18 and 12 synergistically upregulate interferon-γ production by murine dendritic epidermal T cells. J Invest Dermatol. (1999) 113:350–4. doi: 10.1046/j.1523-1747.1999.00697.x 10469333

[42] Wencker M Turchinovich G Di Marco Barros R Deban L Jandke A Cope A. Innate-like T cells straddle innate and adaptive immunity by altering antigen-receptor responsiveness. Nat Immunol. (2014) 15:80–7. doi: 10.1038/ni.2773 PMC6485477 24241693

[43] Omori M Yamashita M Inami M Ukai-Tadenuma M Kimura M Nigo Y. CD8 T cell-specific downregulation of histone hyperacetylation and gene activation of the IL-4 gene locus by ROG, repressor of GATA. Immunity. (2003) 19:281–94. doi: 10.1016/s1074-7613(03)00210-3 12932361

[44] Wei G Wei L Zhu J Zang C Hu-Li J Yao Z. Global mapping of H3K4me3 and H3K27me3 reveals specificity and plasticity in lineage fate determination of differentiating CD4(+) T cells. Immunity. (2009) 30:155–67. doi: 10.1016/j.immuni.2008.12.009 PMC2722509 19144320

[45] Schmolka N Serre K Grosso AR Rei M Pennington DJ Gomes AQ. Epigenetic and transcriptional signatures of stable versus plastic differentiation of proinflammatory γδ T cell subsets. Nat Immunol. (2013) 14:1093–100. doi: 10.1038/ni.2702 PMC4834994 23995235

[46] Moon H Na HY Chong KH Kim TJ. P2X(7) receptor-dependent ATP-induced shedding of CD27 in mouse lymphocytes. Immunol Lett. (2006) 102:98–105. doi: 10.1016/j.imlet.2005.08.004 16207496

[47] Minagawa M Ito A Shimura H Tomiyama K Ito M Kawai K. Homogeneous epithelial γδ T cell repertoire of the skin is shaped through peripheral selection. J Dermatol Sci. (2001) 25:150–5. doi: 10.1016/s0923-1811(00)00119-5 11164711

[48] Zhang B Wu J Jiao Y Bock C Dai M Chen B. Differential requirements of TCR signaling in homeostatic maintenance and function of dendritic epidermal T cells. J Immunol. (2015) 195:4282–91. doi: 10.4049/jimmunol.1501220 PMC4610875 26408667

[49] Sudo K Todoroki T Ka Y Takahara K. Vγ5Vδ1 TCR signaling is required to different extents for embryonic versus postnatal development of DETCs. Int Immunol. (2022) 34:263–76. doi: 10.1093/intimm/dxac001 35031803

[50] Hara H Kishihara K Matsuzaki G Takimoto H Tsukiyama T Tigelaar RE. Development of dendritic epidermal T cells with a skewed diversity of γδTCRs in Vδ1-deficient mice. J Immunol. (2000) 165:3695–705. doi: 10.4049/jimmunol.165.7.3695 11034374

[51] Kisielow J Kopf M Karjalainen K. SCART scavenger receptors identify a novel subset of adult γδ T cells. J Immunol. (2008) 181:1710–6. doi: 10.4049/jimmunol.181.3.1710 18641307

[52] Sumaria N Roediger B Ng LG Qin J Pinto R Cavanagh LL. Cutaneous immunosurveillance by self-renewing dermal γδ T cells. J Exp Med. (2011) 208:505–18. doi: 10.1084/jem.20101824 PMC3058585 21339323

[53] Mendoza MC Er EE Blenis J. The Ras-ERK and PI3K-mTOR pathways: cross-talk and compensation. Trends Biochem Sci. (2011) 36:320–8. doi: 10.1016/j.tibs.2011.03.006 PMC3112285 21531565

[54] Lopes N McIntyre C Martin S Raverdeau M Sumaria N Kohlgruber AC. Distinct metabolic programs established in the thymus control effector functions of γδ T cell subsets in tumor microenvironments. Nat Immunol. (2021) 22:179–92. doi: 10.1038/s41590-020-00848-3 PMC7610600 33462452

[55] Argüello RJ Combes AJ Char R Gigan JP Baaziz AI Bousiquot E. SCENITH: a flow cytometry-based method to functionally profile energy metabolism with single-cell resolution. Cell Metab. (2020) 32:1063–75. doi: 10.1016/j.cmet.2020.11.007 PMC8407169 33264598

[56] Maneechotesuwan K Xin Y Ito K Jazrawi E Lee KY Usmani OS. Regulation of Th2 cytokine genes by p38 MAPK-mediated phosphorylation of GATA-3. J Immunol. (2007) 178:2491–8. doi: 10.4049/jimmunol.178.4.2491 17277157

[57] Furusawa J Moro K Motomura Y Okamoto K Zhu J Takayanagi H. Critical role of p38 and GATA3 in natural helper cell function. J Immunol. (2013) 191:1818–26. doi: 10.4049/jimmunol.1300379 PMC3765427 23851685

[58] Salvador JM Mittelstadt PR Guszczynski T Copeland TD Yamaguchi H Appella E. Alternative p38 activation pathway mediated by T cell receptor-proximal tyrosine kinases. Nat Immunol. (2005) 6:390–5. doi: 10.1038/ni1177 15735648

[59] McKenzie DR Hart R Bah N Ushakov DS Munoz-Ruiz M Feederle R. Normality sensing licenses local T cells for innate-like tissue surveillance. Nat Immunol. (2022) 23:411–22. doi: 10.1038/s41590-021-01124-8 PMC8901436 35165446

[60] Yang Q Liu X Liu Q Guan Z Luo J Cao G. Roles of mTORC1 and mTORC2 in controlling γδ T1 and γδ T17 differentiation and function. Cell Death Differ. (2020) 27:2248–62. doi: 10.1038/s41418-020-0500-9 PMC7308385 32001780

[61] Mills RE Taylor KR Podshivalova K McKay DB Jameson JM. Defects in skin γδ T cell function contribute to delayed wound repair in rapamycin-treated mice. J Immunol. (2008) 181:3974–83. doi: 10.4049/jimmunol.181.6.3974 PMC2547144 18768852

[62] Bai Y Xu R Zhang X Zhang X Hu X Li Y. Differential role of rapamycin in epidermis-induced IL-15-IGF-1 secretion via activation of Akt/mTORC2. Cell Physiol Biochem. (2017) 42:1755–68. doi: 10.1159/000479443 28746918

[63] Itohara S Mombaerts P Lafaille J Iacomini J Nelson A Clarke AR. T cell receptor δ gene mutant mice: Independent generation of αβ T cells and programmed rearrangements of γδ TCR genes. Cell. (1993) 72:337–48. doi: 10.1016/0092-8674(93)90112-4 8381716

[64] Carpino N Thierfelder WE Chang MS Saris C Turner SJ Ziegler SF. Absence of an essential role for thymic stromal lymphopoietin receptor in murine B-cell development. Mol Cell Biol. (2004) 24:2584–92. doi: 10.1128/MCB.24.6.2584-2592.2004 PMC355866 14993294

[65] Uchida Y Kawai K Ibusuki A Kanekura T. Role for E-cadherin as an inhibitory receptor on epidermal γδ T cells. J Immunol. (2011) 186:6945–54. doi: 10.4049/jimmunol.1003853 21562159

